# Determinants of preterm prelabor rupture of fetal membrane among pregnant women in Ethiopia: A systematic review and meta-analysis

**DOI:** 10.1371/journal.pone.0311151

**Published:** 2024-11-08

**Authors:** Habtamu Geremew, Mohammed Ahmed Ali, Mulat Belay Simegn, Eyasu Bamlaku Golla, Alegntaw Abate, Smegnew Gichew Wondie, Hawi Kumbi, Mitku Mammo Taderegew, Werkneh Melkie Tilahun

**Affiliations:** 1 College of Health Science, Oda Bultum University, Chiro, Ethiopia; 2 Department of Midwifery, College of Health Science, Oda Bultum University, Chiro, Ethiopia; 3 Department of Public Health, College of Medicine and Health Science, Debre Markos University, Debre Markos, Ethiopia; 4 Department of Medical Laboratory Science, College of Health Science, Oda Bultum University, Chiro, Ethiopia; 5 Department of Human Nutrition, College of Medicine and Health Science, Mizan Tepi University, Mizan Aman, Ethiopia; 6 Department of Laboratory, Adama Hospital Medical College, Adama, Ethiopia; 7 Department of Biomedical Sciences, College of Medicine and Health Sciences, Wolkite University, Wolkite, Ethiopia; Dicle University, TÜRKIYE

## Abstract

**Introduction:**

Ethiopia is one of the countries where persistently high neonatal and maternal mortalities are reported. Preterm prelabor rupture of membrane (PPROM) plays an important contribution to these high mortalities. However, there is a paucity of comprehensive evidence about the epidemiology of PPROM in Ethiopia. Therefore, this systematic review was conducted to assess the pooled prevalence and determinants of PPROM among pregnant women in Ethiopia.

**Methods:**

A systematic review and meta-analysis were conducted following the PRISMA guideline. Relevant literatures were searched on African Journals Online (AJOL), PubMed, Scopus, Epistemonikos, CINAHL, Cochrane Library and gray literature. All statistical analyses were performed using STATA 17 software. The random effect meta-analysis model was employed to summarize the pooled estimates. Heterogeneity between included studies was evaluated using I^2^ statistic. Egger’s regression test and Begg’s correlation test were employed to assess publication bias, in conjunction with funnel plot. Besides, the non-parametric trim-and-fill analysis, sensitivity analysis, subgroup analysis and meta-regression were also performed.

**Results:**

A total of 13 original studies with 24,386 participants were considered in this systematic review. The pooled prevalence of PPROM was 6.58% (95% CI: 5.36, 7.79). Urinary tract infection (OR: 3.44; 95% CI: 1.81, 6.53), abnormal vaginal discharge (OR: 4.78; 95% CI: 2.85, 8.01), vaginal bleeding (OR: 2.04; 95% CI: 1.03, 4.06), history of PROM (OR: 4.64; 95% CI: 2.71, 7.95), history of abortion (OR: 3.06; 95% CI: 1.71, 5.46), malnutrition (OR: 5.24; 95% CI: 2.63, 10.44), anemia (OR: 3.97; 95% CI: 2.01, 7.85) and gestational diabetes (OR: 5.08; 95% CI: 1.93, 13.36) were significantly associated with PPROM.

**Conclusion:**

This meta-analysis found a high prevalence of PPROM in Ethiopia. Urinary tract infection, abnormal vaginal discharge, vaginal bleeding, history of PROM, history of abortion, malnutrition, anemia and gestational diabetes were risk factors for PPROM. Prevention and control of antenatal infections and malnutrition are highly recommended to reduce the magnitude of PPROM in Ethiopia. Additionally, healthcare providers should emphasize the identified risk factors.

**Protocol registration number:**

CRD42024536647.

## Introduction

Prelabor rupture of the membrane (PROM) refers to the spontaneous rupture of fetal membrane before the onset of true labor [1]. Rupture of fetal membrane that occurs before 37 completed weeks of gestation is called preterm prelabor rupture of membranes (PPROM) [1, 2]. The fetal membrane is composed of two separate layers namely, the chorion, which forms the outer feto-maternal interface barrier and the amnion, which makes the uterine cavity’s innermost lining; this innermost membrane is contiguous with amniotic fluid [3]. Amniochorion offers protection to the fetus; thus, defective early rupture of this membrane jeopardizes both maternal and neonatal outcomes [4].

Globally, PPROM affects about 3% of pregnancies [1, 5]. The burden of PROM is greatest in low and middle-income countries, where most prematurity-related child mortalities occur [6]. For instance, a cross-sectional study in Cameroon reported that 4.9% of pregnancies were complicated with PPROM [7]. Another institutional-based survey in Uganda found that 7.5% of pregnant women suffered from PPROM [8]. In Ethiopia, few previous studies reported the magnitude of PPROM, and it varies between 13.7% and 6.6% [2, 9].

PPROM has various complications affecting both the mother and her fetus [10]. It precedes one-third of preterm deliveries [2]. Prelabor membrane rupture causes about 15% of perinatal deaths and 33% of perinatal illnesses [11]. Some of the common neonatal morbidities following PPROM include respiratory distress syndrome, necrotizing enterocolitis, intraventricular hemorrhage and hyperbilirubinemia [4, 12]. Mothers are also affected by complications like infection, disseminated intravascular coagulation, cervical incompetence, cord prolapse, placenta abruption and postpartum hemorrhage [4, 13]. PPROM also impacts the economy through medical expenses, hospitalization, lost productivity and expense to the healthcare providers [14].

The specific cause that leads to PPROM has not been documented so far; however, previous studies have identified different risk factors including poor socio-economic status, short inter-pregnancy interval, anemia, malnutrition, previous history of abortion, gestational diabetes mellitus, abnormal vaginal discharge, urinary tract infection and history of premature rupture of the membrane [10, 15, 16].

Ethiopia is one of the countries where persistently high neonatal and maternal mortalities are reported [17]. PPROM plays an important contribution to these high mortalities. The Ministry of Health has implemented different strategies like the expansion of advanced obstetric and neonatal care, and development of training manuals and guidelines to equip medical personnel with the necessary skills to manage and refer obstetric emergencies including PPROM [18]. However, the problem continues to be a grave public health concern [2]. There is also a paucity of dependable evidence to inform clinical care and management of PPROM in Ethiopia. There exist few studies that investigated the prevalence and determinants of PPROM. However, their findings were inconsistent and difficult to conclude. Therefore, this review was conducted to summarize the prevalence and determinants of PPROM among pregnant women in Ethiopia.

## Methods and materials

### Reporting

The review protocol was registered on the International Prospective Register of Systematic Reviews (PROSPERO) database with registration number: CRD42024536647. The Preferred Reporting Items for Systematic Reviews and Meta-Analyses (PRISMA) checklist was strictly followed to report this analysis [19], (S1 Checklist).

### Search strategy

We employed a comprehensive web-based search on African Journals Online (AJOL), PubMed, Scopus, Epistemonikos, CINAHL and Cochrane Library. Other gray literature sources like Google and online repositories of Ethiopian university repositories were also examined. In addition, the reference lists of pertinent articles were also scrutinized to identify additional reports. Two researchers (HG and MBS) independently searched the databases from May 1 to 30, 2024. The following keywords were used to conduct the literature search: (prevalence OR incidence OR Magnitude OR Epidemiology) AND (“preterm premature rupture of membrane” OR “preterm prelabor rupture of membrane” OR “prelabor rupture of membrane” OR “premature rupture of membrane” OR “premature rupture of fetal membrane” OR PPROM OR PROM) AND (“Associated factor*” OR Determinant* OR Factor* OR “Risk factor*”) AND Ethiopia.

### Eligibility criteria

Eligible studies were identified based on CoCoPop (condition, context, and population) mnemonic [20]. Consequently, all studies published in the English language and reported the number and/or prevalence and/or associated factors of PPROM among pregnant women in Ethiopia and fulfill the following conditions were included in this review.

Population/Participant: pregnant women.

Condition: PPROM.

Context: Studies conducted only in Ethiopia setting.

Study design: All observational studies (Cohort, Case-control and Cross-sectional). Nevertheless, studies that reported both preterm and term PROM compositely, case reports, editorial letters, clinical trials, duplicate studies, abstracts without full text and qualitative studies without outcome of interest were excluded from this analysis.

### Study outcomes and measurements

The primary outcome of this analysis was the prevalence of PPROM, and it was defined as the spontaneous rupture of fetal membrane after fetal viability (>28 weeks of gestation) but before 37 completed weeks of gestation and prior to the onset of true labor [2]. We also sought to identify factors associated with PPROM among pregnant women in Ethiopia.

Malnutrition: a woman was considered as malnourished if her mid-upper arm circumference (MUAC) was less than 23 centimeters [2, 21].

Anemia: a woman was considered as anemic if her blood hemoglobin concentration was below 11 g/dl [2].

### Study selection, quality assessment, and data extraction

Identified studies were exported to reference management software (Endnote version X7.2), where duplicate records were eliminated. The remaining studies were then assessed by title and abstract. For records found to be pertinent by title and abstract, a full-text review was performed against the pre-specified inclusion/exclusion criteria to identify potential articles to be included in this systematic review.

The quality of the included studies was assessed using the Joanna Brigg’s Institute (JBI) quality assessment checklist [22], (S1 File). Two authors of this review (HG & WMT) evaluated the quality of the studies independently and inconsistencies were resolved by involving a third author (MAA).

Relevant data were extracted into an Excel spreadsheet. A standardized data extraction form which was developed by considering the JBI guide for data extraction and synthesis was used [23]. The retrieved data include last name of the primary author, year of publication, study area, study design, sample size (total participants) and number/prevalence of PPROM. Additionally, information about predictor variables that were found to be significant determinants in one of the included studies and also reported in at least one other study were extracted into separate Excel spreadsheets.

### Analysis

After extracting into an Excel spreadsheet, the data were exported to STATA version 17 software for further statistical analysis. The general characteristics of the included studies are presented in tables. The pooled prevalence estimate was calculated using the prevalence and standard errors of included studies. The random effects meta-analysis model was employed to estimate the pooled effect size, and estimates were depicted by forest plots. Heterogeneity between studies was assessed using the I^2^ statistics and it was considered as high, moderate, or low when I^2^ test statistics results were 75%, 50%, and 25% respectively [24]. Egger’s regression test and Begg’s correlation test were employed to assess publication bias, in conjunction with funnel plot. Besides, the non-parametric trim-and-fill analysis, sensitivity analysis, subgroup analysis and meta-regression were also performed. Furthermore, almost all the included studies had a 100% response rate, and no significant data were missing. As a result, no additional statistical methods were employed to this end.

## Results

### Identification of records

**Fig 1:**
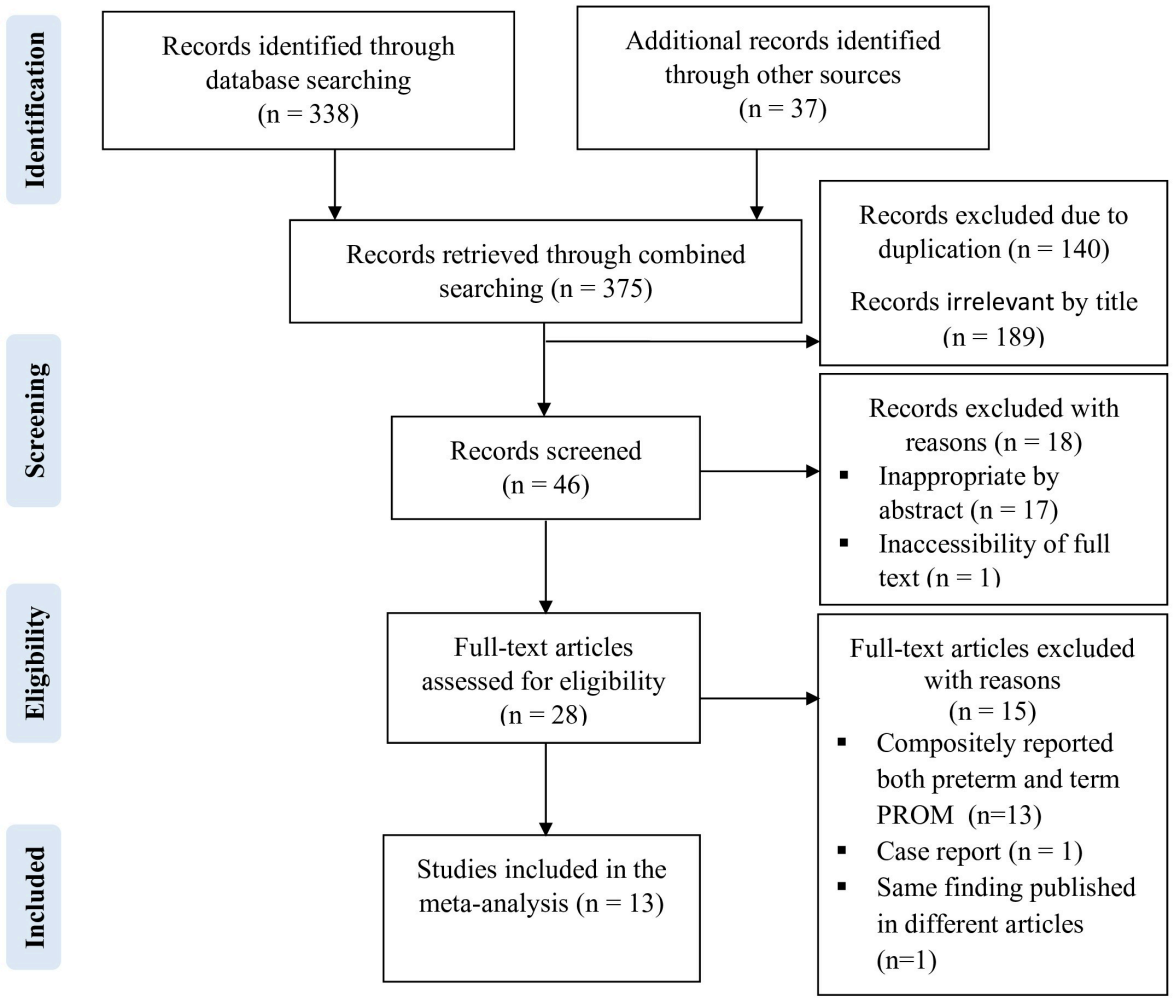
PRISMA flow chart for studies identified, screened and included.

The combined literature search yielded 375 records. One hundred forty duplicates were removed. Then, the titles of 235 records were assessed, of which 189 were irrelevant. Finally, after reviewing 46 abstracts and 28 full text articles, 13 studies were found to be suitable for inclusion in this meta-analysis (Fig 1) (S2 File).

### Study characteristics

A total of 13 original studies with 24,386 participants were considered in this systematic review. The sample size of the included studies ranged between 197 [9] and 8283 [25]. Except for one community-based study [15], all studies were conducted within health facilities. Methodologically, three studies employed cohort study design [15, 26, 27], while the remaining ten studies employed cross-sectional study design [2, 9, 16, 21, 25, 28–32]. The prevalence of PPROM as reported by primary studies varies between 1.34% and 22.76% (Table 1) (S3 File).

**Table 1:** Characteristics of included studies.

Last name of the first author	Year of publication	Study area	Study design	Setting	Sample size	Prevalence of PPROM	Risk of bias
Abaynew [16]	2021	Debre Markos	Cross-sectional	Facility based	425	14.35%	Low risk
Addisu [2]	2020	Debre Tabor	Cross-sectional	Facility based	424	13.68%	Low risk
Argaw [9]	2021	Wolkite	Cross-sectional	Facility based	197	6.60%	Low risk
Gutema [30]	2023	Ambo	Cross-sectional	Facility based	391	22.76%	Low risk
Sirak [25]	2014	Addis Ababa	Cross-sectional	Facility based	8283	1.34%	Low risk
Tsegaye [21]	2023	Harar	Cross-sectional	Facility based	449	14.25%	Low risk
Jena [15]	2022	Hadiya zone	Cohort	Community-based	2548	1.96%	Low risk
Abebe [26]	2023	Addis Ababa	Cohort	Facility based	7235	2.21%	Low risk
Diriba [32]	2022	West Guji Zone	Cross-sectional	Facility based	407	4.18%	Low risk
Telayneh [29]	2023	Debre Markos	Cross-sectional	Facility based	315	11.11%	Low risk
Tolera [31]	2022	Dire Dawa	Cross-sectional	Facility based	392	4.59	Low risk
Wolde [28]	2024	Harar	Cross-sectional	Facility based	424	6.84%	Low risk
Segni [27]	2017	Jimma	Cohort	Facility based	2896	1.45%	Low risk

### Prevalence of PPROM

**Fig 2:**
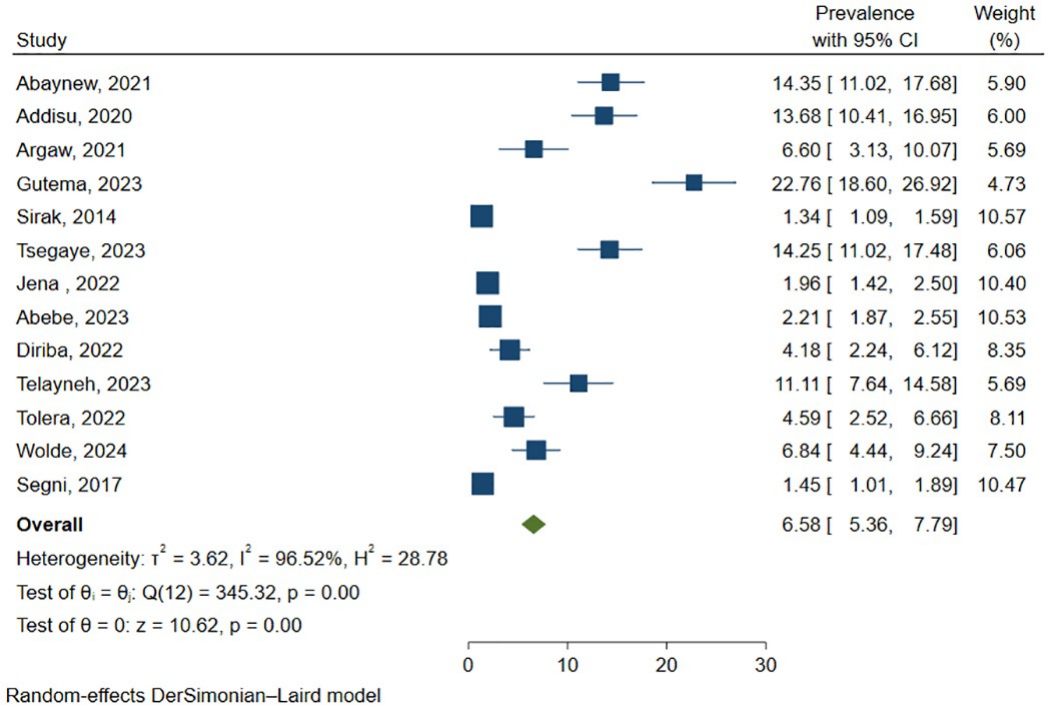
Pooled prevalence of PPROM in Ethiopia.

In this systematic review and meta-analysis, the pooled prevalence of PPROM among pregnant women in Ethiopia after combining reports of 13 primary studies involving 24,386 participants was 6.58% (95% CI: 5.36, 7.79) (Fig 2).

### Heterogeneity and sensitivity analysis

**Fig 3:**
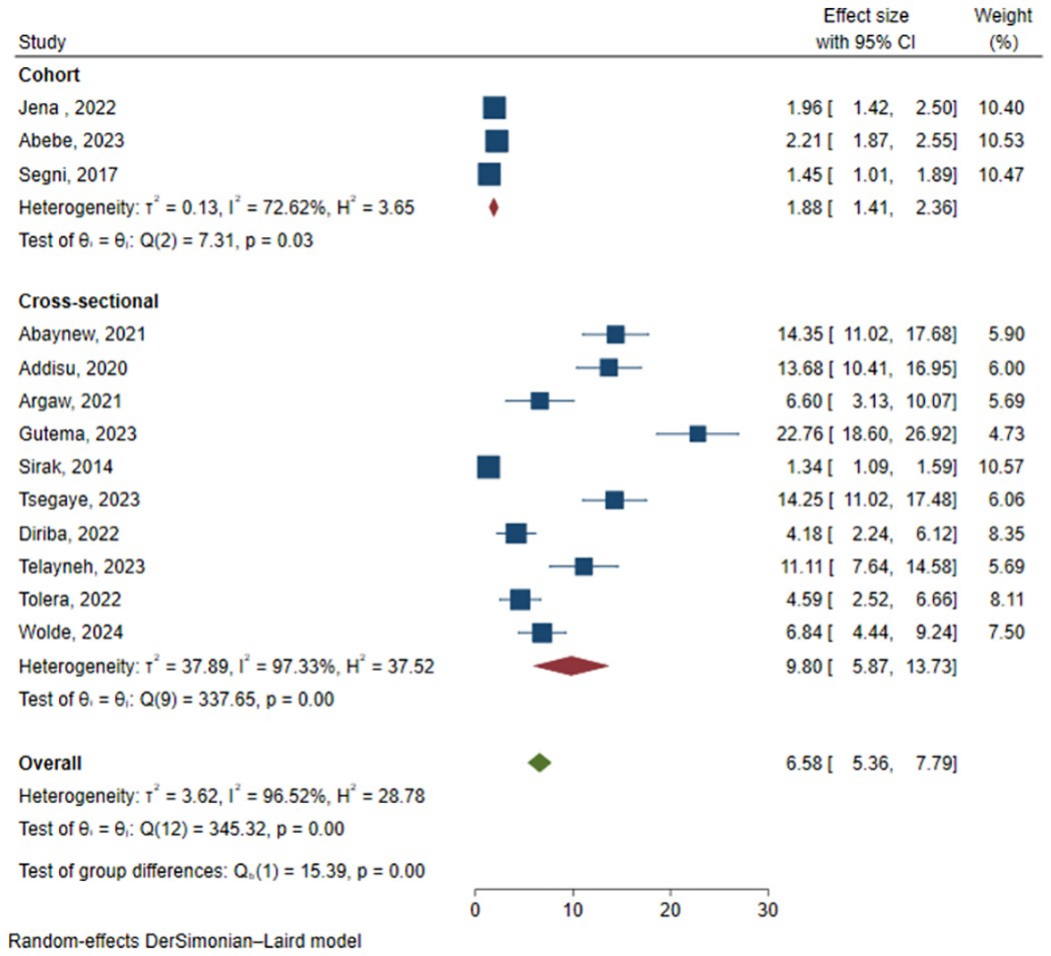
Sub-group analysis of prevalence of PPROM by study design.

**Fig 4:**
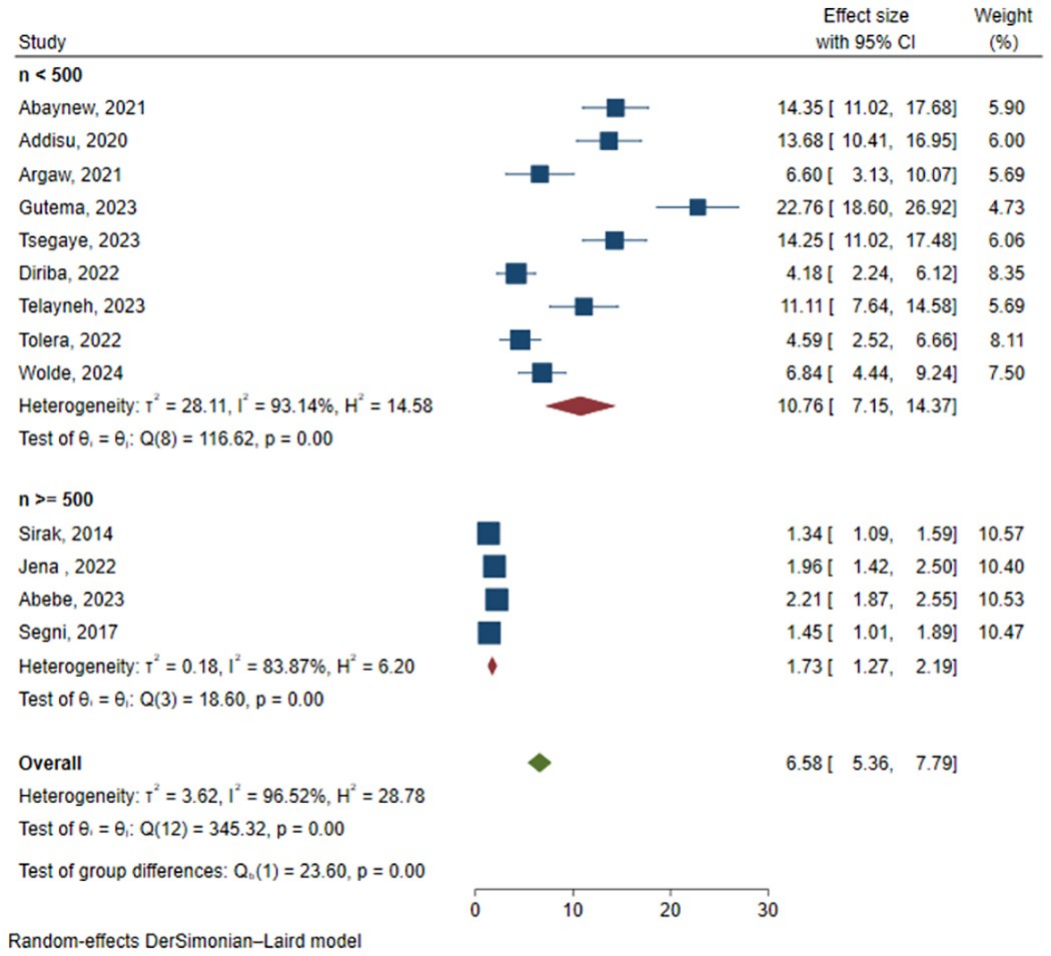
Sub-group analysis of prevalence of PPROM by sample size.

The I^2^ statistic indicated a statistically significant substantial heterogeneity between included studies (96.52%, p-value<0.01). In light of this, the random effect meta-analysis model (DerSimonian and Laird method) was employed to pool the estimates. To further explore the between-study heterogeneity, sub-group analysis was conducted by study design (cross-sectional and cohort) and sample size (<500 and ≥500), and there was a statistically significant difference in the prevalence of PPROM between the groups of both study design (Fig 3) and sample size (Fig 4). This finding was further strengthened by the random effect meta-regression output which found a statistically significant effect of sample size on between study heterogeneity of PPROM prevalence in Ethiopia (Table 2).

**Table 2:** Meta-regression analysis of factors for the heterogeneity of PPROM prevalence in Ethiopia.

Source of heterogeneity	Coefficient	Standard error	T	P > |t|	95% Confidence Interval
Sample size	-0.0011279	0.0004017	-2.81	0.019	-0.002023, -0.0002328
Publication year	0.2242108	0.3959688	0.57	0.584	-0.6580626, 1.106484
Constant	-443.3688	800.735	-0.55	0.592	-2227.517, 1340.78

**Fig 5:**
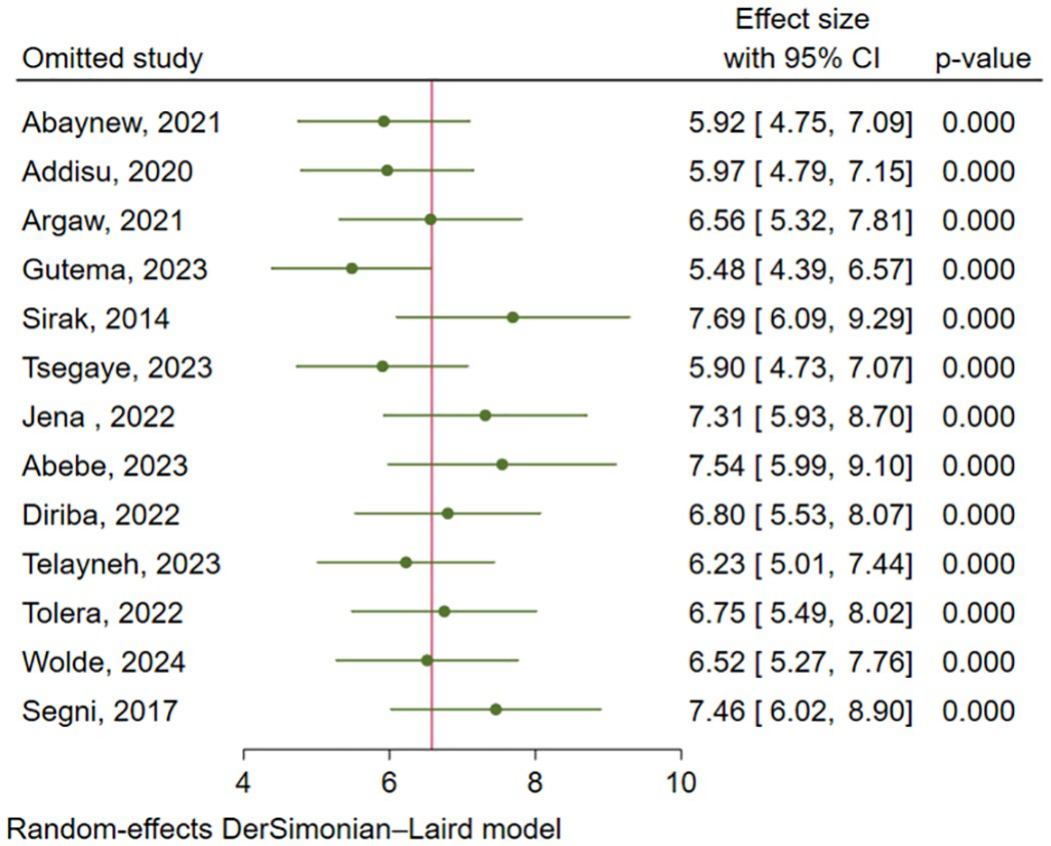
Sensitivity analysis for prevalence of PPROM in Ethiopia.

The leave-one-out sensitivity analysis was performed to ensure the stability of the overall effect size estimate, and the result showed that the pooled estimate was not unduly influenced by a single study. Hence, the point estimate of PPROM for each omitted analysis lies within the CI of the combined analysis (Fig 5).

### Publication bias

**Fig 6:**
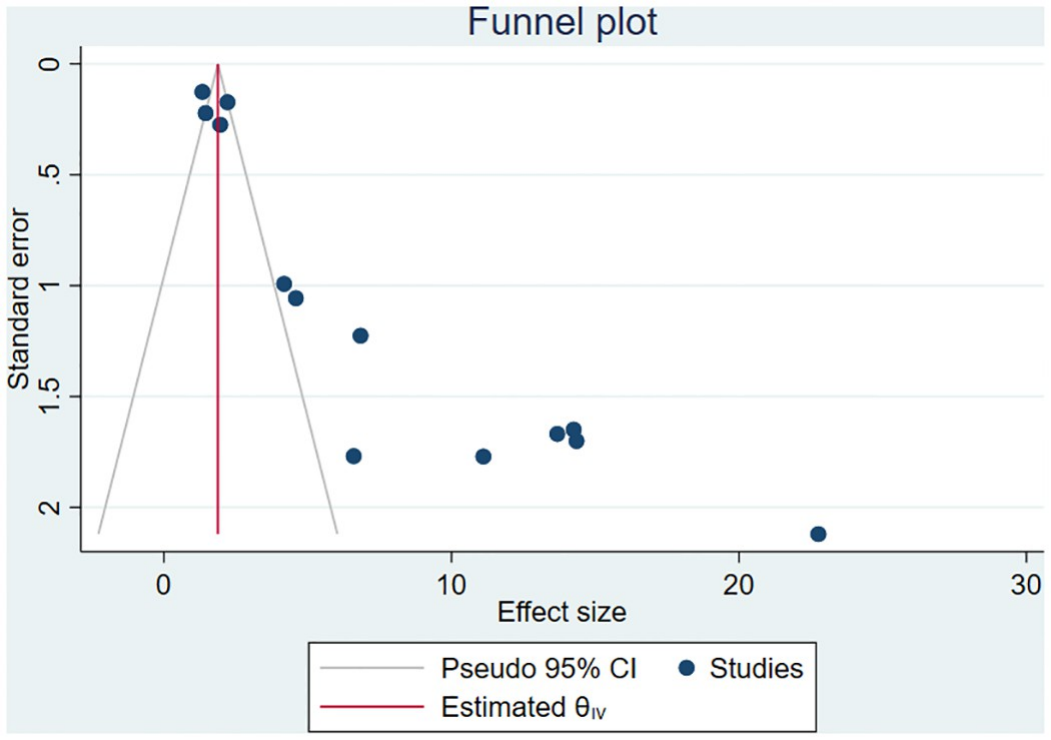
Funnel plot, evaluating publication bias for prevalence of PPROM in Ethiopia.

**Fig 7:**
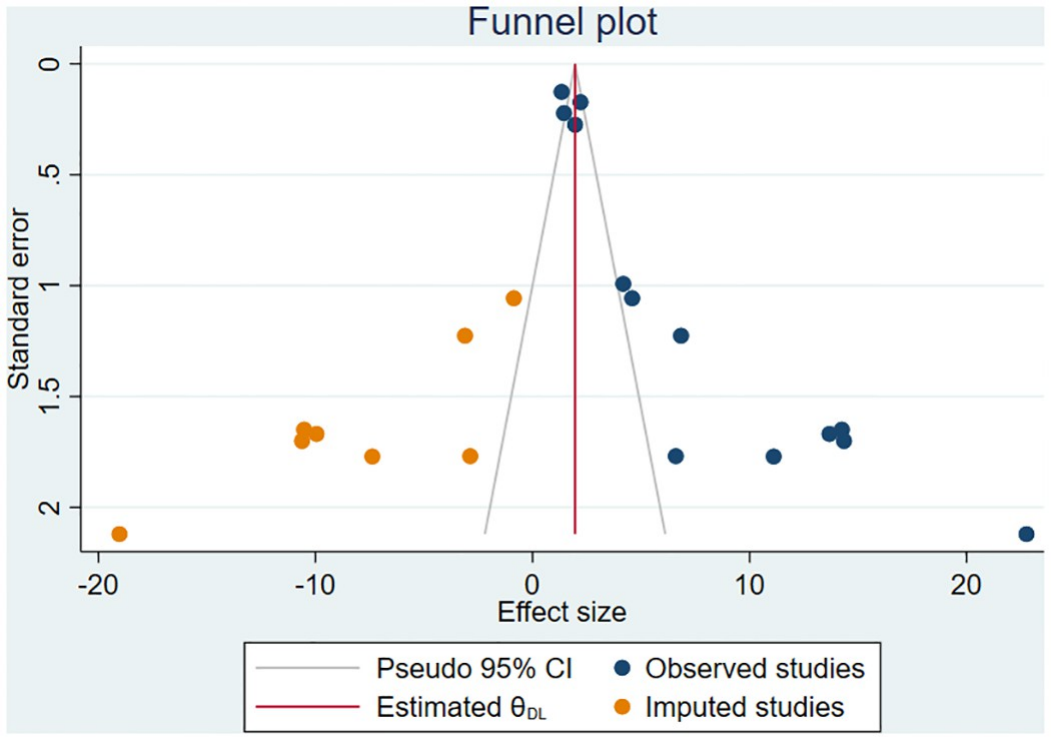
Funnel plop, after the trim-and-fill analysis for prevalence of PPROM in Ethiopia.

Small study effect was investigated using both the subjective (funnel plot) and the objective (Begg and Egger’s tests) methods. As a result, the asymmetrical funnel plot depicted the presence of publication bias (Fig 6). This finding was further corroborated by the significant Egger’s regression test (p-value<0.0001) and Begg’s correlation test (p-value = 0.0008). Considering this significant publication bias, the non-parametric trim-and-fill analysis was conducted. After incorporating eight additional imputed studies using the run R0 estimator (Fig 7), the adjusted pooled prevalence of PPROM was found to be 1.96% (95% CI: 0.71, 3.20).

### Determinants of PPROM

Data about variables that were found significant predictors of PPROM in one of the included studies and also reported in at least one other study with or without significant association were extracted into to separate Excel spreadsheet. As a result, data about eight independent variables (urinary tract infection, abnormal vaginal discharge, vaginal bleeding, history of PROM, history of abortion, malnutrition, anemia and gestational diabetes) were extracted and analyzed separately. Surprisingly, all of the variables were found to be significant predictors of PPROM. Accordingly, women who had urinary tract infections were 3.44 (95% CI: 1.81, 6.53) times more likely to develop PPROM than their counterparts. The risk of PPROM was 4.78 (2.85, 8.01) times higher among pregnant women who had abnormal vaginal discharge as compared to pregnant women without abnormal vaginal discharge. Similarly, women who had vaginal bleeding were 2.04 (95% CI: 1.03, 4.06) times more likely to suffer from PPROM than their counterparts. Additionally, history of PROM (OR: 4.64; 95% CI: 2.71, 7.95), history of abortion (OR: 3.06; 95% CI: 1.71, 5.46), malnutrition (OR: 5.24; 95% CI: 2.63, 10.44), anemia (OR: 3.97; 95% CI: 2.01, 7.85) and gestational diabetes (OR: 5.08; 95% CI: 1.93, 13.36) were also significantly associated with PPROM (Table 3).

**Table 3:** Determinants of PPROM among pregnant women in Ethiopia.

Variables	Exposed	Comparator	Included studies	Total participants	OR (95% CI)	Heterogeneity (p-value)
Urinary tract infection	Yes	No	4	1589	3.44 (1.81, 6.53)	0.002
Abnormal vaginal discharge	Yes	No	2	849	4.78 (2.85, 8.01)	0.280
Vaginal bleeding	Yes	No	4	1493	2.04 (1.03, 4.06)	0.027
History of PROM	Yes	No	4	1689	4.64 (2.71, 7.95)	0.048
History of abortion	Yes	No	3	1071	3.06 (1.71, 5.46)	0.168
Malnutrition	Yes	No	3	1298	5.24 (2.63, 10.44)	0.014
Anemia	Yes	No	3	1012	3.97 (2.01, 7.85)	0.257
Gestational diabetes	Yes	No	2	588	5.08 (1.93, 13.36)	0.158

## Discussion

This study aimed to assess the prevalence and determinants of PPROM among pregnant women in Ethiopia. Correspondingly, the pooled prevalence estimate of PPROM among pregnant women in Ethiopia was 6.58% (95% CI: 5.36, 7.79). Urinary tract infection, abnormal vaginal discharge, vaginal bleeding, history of PROM, history of abortion, malnutrition, anemia and gestational diabetes were the significant determinants of PPROM.

The prevalence of PPROM in this study was lower than has been reported in a recent meta-analysis by Santos et al, 9% [33]. This might be due to the difference in study participants. The study by Santos and his colleagues was a meta-analysis of studies conducted among systemic lupus erythematous patients, and it is evidenced elsewhere that systemic lupus erythematous significantly increases the risk of PPROM [34]. On the other hand, our prevalence estimate was higher than studies conducted in Canada and the United States, 2–3% [5, 35]. This discrepancy could be attributed to the high rate of antenatal infection and other risk factors in Ethiopia [5, 36].

Sub-group analysis showed that there was a significantly higher prevalence of PPROM among studies with a sample size of less than 500 participants as compared to studies with greater than or equal to 500 participants. This is also indicated in the random effect meta-regression analysis which revealed an inverse association between sample size and PPROM prevalence. This might be due to the imprecision of estimates with a lower sample size [37]. In addition, all of the studies with smaller sample sizes were cross-sectional studies while studies with larger sample sizes were cohort studies, and it is known that cross-sectional studies usually result in higher magnitude than cohort studies [38], as also detected in this meta-analysis.

The odds of PPROM were 3.44 times higher among pregnant women with urinary tract infections than women without urinary tract infections. This finding is in agreement with previous studies from Cameroon [7], and Israel [39]. One possible explanation for this association could be due to ascending infection passing through the cervical canal [8]. Pregnant women who had abnormal vaginal discharge were 4.78 times more likely to experience PPROM than their counterparts. This might be due to the reduction of fetal membrane tightness with genital infections [40]. Similarly, pregnant women who had vaginal bleeding were 2.04 times more likely to suffer from PPROM as compared to their counterparts. The possible explanation for this might be due to the increased thrombin production, which stimulates the secretion of matrix metalloproteinases that can degrade the extracellular matrix of the chorioamniotic membranes [41].

In this meta-analysis, pregnant women who had a history of PROM were 4.64 times more likely to experience PPROM than women without such a history. Likewise, pregnant women who had a history of abortion were 3.06 times more likely to suffer from PPROM as compared to their counterparts. These associations were also documented in previous reports [18, 35], and might be due to preexisting untreated infections, cervical dysfunction or the reappearance of risk factors among pregnant women with such history [5, 42].

The risk of PPROM was 5.24 times higher among pregnant women who were malnourished than women who were not malnourished. This could be due to the malnutrition-related lack of micronutrients like vitamin C and vitamin E, thereby leading to diminished collagen synthesis and tearing of the fetal membrane [43, 44]. The odds of PPROM were also 3.97 times higher among pregnant women with anemia than their counterparts. This might be due to the decreased tissue oxygenation, potentially raising the risk of PPROM due to hypoxia [45]. Pregnant women with gestational diabetes were 5.08 times more likely to develop PPROM than women without gestational diabetes. This finding is supported by the results of a prospective cohort study in Canada [35] and might be due to the overstretching of the fetal membrane because of the high rate of polyhydramnios and macrosomia in gestational diabetes [46].

The findings of this literature review should be interpreted with due consideration of the following limitations. There was a statistically significant heterogeneity between the included studies. In addition, assessment of publication bias indicated the presence of small study effect that substantially affected the prevalence of PPROM.

## Conclusion

This meta-analysis found a high prevalence of PPROM in Ethiopia. Urinary tract infection, abnormal vaginal discharge, vaginal bleeding, history of PROM, history of abortion, malnutrition, anemia and gestational diabetes were significant risk factors for PPROM. Prevention and control of antenatal infections and malnutrition are highly recommended to reduce the magnitude of PPROM in Ethiopia. Additionally, healthcare providers should emphasize the identified risk factors.

## Supporting information

S1 Checklist PRISMA 2020 checklist. (DOCX)

S1 File Risk of bias assessment for included studies. (DOCX)

S2 File Studies excluded after full text review. (DOCX)

S3 File Extracted data with name of extractors and date of extraction. (DOCX)
