# Supplementary material for: Determinants of preterm prelabor rupture of fetal membrane among pregnant women in Ethiopia: A systematic review and meta-analysis
Source: PLoS One. 2024 Nov 8;19(11):e0311151. doi: 10.1371/journal.pone.0311151 (PMC11548779; doi:10.1371/journal.pone.0311151)
Supplement: S1 File — (DOCX) [file pone.0311151.s002.docx]

**Risk of bias assessment for included studies using the JBI quality assessment checklist**

1. Quality assessment result of cohort studies

| Study | **JBI Critical Appraisal Checklist** | | | | | | | | |  |  | Overall risk of bias |
| --- | --- | --- | --- | --- | --- | --- | --- | --- | --- | --- | --- | --- |
|  | Were the two groups similar and recruited from the  same population? | Were the exposures measured similarly to assign  people to both exposed and unexposed groups? | Was the exposure measured in a valid and reliable  way? | Were confounding factors identified? | Were strategies to deal with confounding factors  stated? | Were the groups/participants free of the outcome  at the start of the study (or at the moment of  exposure)? | Were the outcomes measured in a valid and reliable  way? | Was the follow up time reported and sufficient to  be long enough for outcomes to occur? | Was follow up complete, and if not, were the  reasons to loss to follow up described and explored? | Were strategies to address incomplete follow up  utilized? | Was appropriate statistical analysis used? |  |
| Jena et al, 2022 | Yes | Yes | Yes | Yes | Yes | Yes | Yes | Yes | Yes | Yes | Yes | Low |
| Abebe et al, 2023 | Yes | Unclear | Yes | Yes | No | Yes | Yes | No | Unclear | Unclear | Yes | Low |
| Segni et al, 2017 | Yes | Unclear | Yes | Yes | Yes | Yes | Yes | No | Unclear | Unclear | Yes | Low |

1. Quality assessment result of cross-sectional studies

| Study | **JBI Critical Appraisal Checklist** | | | | | | | | | Overall risk of bias |
| --- | --- | --- | --- | --- | --- | --- | --- | --- | --- | --- |
|  | Was the sample frame appropriate to address the target population? | Were study participants sampled in an appropriate way? | Was the sample size adequate? | Were the study subjects and the setting described in detail? | Was the data analysis conducted with sufficient coverage of the identified sample? | Were valid methods used for the identification of the condition? | Was the condition measured in a standard, reliable way for all participants? | Was there appropriate statistical analysis? | Was the response rate adequate, & if not, was the low response rate managed appropriately? |  |
| Abaynew et al, 2021 | Yes | Yes | Yes | Yes | Yes | Yes | Yes | Yes | Yes | Low |
| Addisu et al, 2020 | Yes | Yes | Yes | Yes | Yes | Yes | Yes | Yes | Yes | Low |
| Argaw et al, 2021 | Yes | Yes | Yes | Yes | Yes | Yes | Yes | Yes | Yes | Low |
| Gutema et al, 2023 | Yes | Yes | Yes | Yes | Yes | Yes | Yes | Yes | Yes | Low |
| Sirak et al, 2014 | Yes | Yes | Unclear | Yes | Yes | Unclear | Unclear | Yes | Yes | Low |
| Tsegaye et a, 2023 | Yes | Yes | Yes | Yes | Yes | Unclear | Unclear | Yes | Yes | Low |
| Diriba et al, 2022 | Yes | Yes | Yes | Yes | Yes | Yes | Yes | Yes | Yes | Low |
| Telayneh et al, 2023 | Yes | Yes | Yes | Yes | Yes | Yes | Yes | Yes | Yes | Low |
| Tolera et al, 2022 | Yes | Yes | Yes | Yes | Yes | Yes | Yes | Yes | Yes | Low |
| Wolde et al, 2024 | Yes | Yes | Yes | Yes | Yes | Yes | Yes | Yes | Yes | Low |
