# Supplementary material for: Determinants of preterm prelabor rupture of fetal membrane among pregnant women in Ethiopia: A systematic review and meta-analysis
Source: PLoS One. 2024 Nov 8;19(11):e0311151. doi: 10.1371/journal.pone.0311151 (PMC11548779; doi:10.1371/journal.pone.0311151)
Supplement: S2 File — (DOCX) [file pone.0311151.s003.docx]

**Studies excluded after full text review**

| **No** | **Study** | **Reason for exclusion** |
| --- | --- | --- |
| 1 | Arusi M, Abdulhakim E, Awol Y, Mosa H. Management of preexisting pelvic organ prolapse in pregnancy complicated with preterm premature rupture of membrane: a case report. Journal of Medical Case Reports. 2023 Jun 9;17(1):252. | Case report |
| 2 | Abebe TA, Nima DD, Mariye YF, Leminie AA. Determinants for perinatal adverse outcomes among pregnant women with preterm premature rupture of membrane: A prospective cohort study. Frontiers in Reproductive Health. 2022 Dec 15;4:1052827. | Same finding published in different articles |
| 3 | Yadeta TA, Egata G, Seyoum B, Marami D. Khat chewing in pregnant women associated with prelabor rupture of membranes, evidence from eastern Ethiopia. Pan African Medical Journal. 2020 May 1;36(1). | Same finding published in different articles |
| 4 | Enjamo M, Deribew A, Semagn S, Mareg M. Determinants of premature rupture of membrane (PROM) among pregnant women in Southern Ethiopia: A case-control study. International journal of women's health. 2022 Mar 31:455-66. | Compositely reported both preterm and term PROM |
| 5 | Habte A, Dessu S, Lukas K. Determinants of premature rupture of membranes among pregnant women admitted to public hospitals in Southern Ethiopia, 2020: A hospital-based case–control study. International journal of women's health. 2021 Jun 22:613-26. | Compositely reported both preterm and term PROM |
| 6 | Assefa NE, Berhe H, Girma F, Berhe K, Berhe YZ, Gebreheat G, Werid WM, Berhe A, Rufae HB, Welu G. Risk factors of premature rupture of membranes in public hospitals at Mekele city, Tigray, a case control study. BMC pregnancy and childbirth. 2018 Dec;18:1-7. | Compositely reported both preterm and term PROM |
| 7 | Alene AA, Mengesha EW, Dagnew GW. Adverse fetal birth outcomes and its associated factors among mothers with premature rupture of membrane in Amhara region, Ethiopia. Plos one. 2024 Apr 1;19(4):e0298319. | Compositely reported both preterm and term PROM |
| 8 | Getnet A, Oljira L, Assefa N, Tiruye G, Figa Z. Determinants of premature rupture of membrane among pregnant women in Harar town, Eastern Ethiopia: A case-control study. Heliyon. 2023 Apr 1;9(4). | Compositely reported both preterm and term PROM |
| 9 | Weldegeorges DA, Welay FT, Mengesha MB, Gebremeskel SG, Kassahun SS, Gebremedhin TS. Exploring the incidence of premature rupture of membrane and its associated factors in public hospitals of eastern zone Tigray region, Ethiopia, 2019. Current Women's Health Reviews. 2022 Feb 1;18(1):140-5. | Compositely reported both preterm and term PROM |
| 10 | Assefa EM, Chane G, Teme A, Nigatu TA. Determinants of prelabor rupture of membrane among pregnant women attending governmental hospitals in Jimma zone, Oromia region, Ethiopia: A multi-center case-control study. Plos one. 2023 Nov 30;18(11):e0294482. | Compositely reported both preterm and term PROM |
| 11 | Yosef ST, Adissu E, Amare M, Ashagire M, Birhane T. Determinants of premature membrane rupture among mothers receiving labor care at different public hospitals in Northeast Ethiopia: An unmatched case control study. | Compositely reported both preterm and term PROM |
| 12 | Ashiko A, Lera T, Kussa S, Geta T, Kassaye G, Tilahun C. Determinants of Prelabor Rupture of Membrane among Pregnant Women Admitted to Hospitals in Wolaita Zone, Southern Ethiopia, 2022. International Journal of Women's Health Care. 2023 Sep 23;8(2):86-94. | Compositely reported both preterm and term PROM |
| 13 | Asaye MM, Gelaye KA, Matebe YH, Lindgren H, Erlandsson K. Effect of fetal malposition, primiparous, and premature rupture of membrane on neonatal Near miss mediated by grade three meconium-stained amniotic fluids and duration of the active first stage of labor: mediation analysis. Plos one. 2023 May 5;18(5):e0285280. | Compositely reported both preterm and term PROM |
| 14 | Abdella MN, Meskelu J, Teklu AM, Bekele D. OBSTETRIC REFERRALS AT SAINT PAUL’S HOSPITAL MILLENNIUM MEDICAL COLLEGE (SPHMMC): PRE-REFERRAL CARE AND APPROPRIATENESS. Ethiopian Journal of Reproductive Health. 2019 Apr 30;11(2):7-. | Compositely reported both preterm and term PROM |
| 15 | Chekol M, Arefaynie M, Mengist A, Guadie M, and Feleke SF. Determinate of Premature Rupture of Membrane among Women Who Gave Birth at Tefera Hailu Memorial Hospital, Northern Ethiopia: Unmatched Case-Control. Open Journal of Public Health, 2023. | Compositely reported both preterm and term PROM |
