# Supplementary material for: Determinants of preterm prelabor rupture of fetal membrane among pregnant women in Ethiopia: A systematic review and meta-analysis
Source: PLoS One. 2024 Nov 8;19(11):e0311151. doi: 10.1371/journal.pone.0311151 (PMC11548779; doi:10.1371/journal.pone.0311151)
Supplement: S3 File — (DOCX) [file pone.0311151.s004.docx]

**Extracted data with name of extractors and date of extraction**

| Study | Study design | Setting | Sample size | Prevalence of PPROM | Date of data extraction | Data extractors | Eligibility for inclusion |
| --- | --- | --- | --- | --- | --- | --- | --- |
| Abaynew et al, 2021 | Cross-sectional | Facility based | 425 | 14.35% | 05/06/2024 G.C | Habtamu Geremew and Mohammed Ahmed Ali | Eligible |
| Addisu et al, 2020 | Cross-sectional | Facility based | 424 | 13.68% | 06/06/2024 G.C | Habtamu Geremew and Mulat Belay Simegn | Eligible |
| Argaw et al, 2021 | Cross-sectional | Facility based | 197 | 6.60% | 07/06/2024 G.C | Habtamu Geremew and Werkneh Melkie Tilahun | Eligible |
| Gutema et al, 2023 | Cross-sectional | Facility based | 391 | 22.76% | 05/06/2024 G.C | Habtamu Geremew and Mohammed Ahmed Ali | Eligible |
| Sirak et al, 2014 | Cross-sectional | Facility based | 8283 | 1.34% | 06/06/2024 G.C | Habtamu Geremew and Mulat Belay Simegn | Eligible |
| Tsegaye et al, 2023 | Cross-sectional | Facility based | 449 | 14.25% | 07/06/2024 G.C | Habtamu Geremew and Werkneh Melkie Tilahun | Eligible |
| Jena et al, 2022 | Cohort | Community-based | 2548 | 1.96% | 05/06/2024 G.C | Habtamu Geremew and Mohammed Ahmed Ali | Eligible |
| Abebe et al, 2023 | Cohort | Facility based | 7235 | 2.21% | 06/06/2024 G.C | Habtamu Geremew and Mulat Belay Simegn | Eligible |
| Diriba et al, 2022 | Cross-sectional | Facility based | 407 | 4.18% | 07/06/2024 G.C | Habtamu Geremew and Werkneh Melkie Tilahun | Eligible |
| Telayneh et al, 2023 | Cross-sectional | Facility based | 315 | 11.11% | 05/06/2024 G.C | Habtamu Geremew and Mohammed Ahmed Ali | Eligible |
| Tolera et al, 2022 | Cross-sectional | Facility based | 392 | 4.59 | 06/06/2024 G.C | Habtamu Geremew and Mulat Belay Simegn | Eligible |
| Wolde et al, 2024 | Cross-sectional | Facility based | 424 | 6.84% | 07/06/2024 G.C | Habtamu Geremew and Werkneh Melkie Tilahun | Eligible |
| Segni et al, 2017 | Cohort | Facility based | 2896 | 1.45% | 05/06/2024 G.C | Habtamu Geremew and Mohammed Ahmed Ali | Eligible |
